# Repeatability and internal consistency of abdominal 2D and 4D PC MR flow measurements

**DOI:** 10.1186/1532-429X-14-S1-W13

**Published:** 2012-02-01

**Authors:** Andrew Wentland, Thomas M Grist, Oliver Wieben

**Affiliations:** 1 Medical Physics, UW - Madison, Madison, WI, USA; 2 Radiology, UW - Madison, Madison, WI, USA

## Background

We have recently demonstrated the benefits of a radially undersampled 4D-MR flow acquisition (PC-VIPR) [1,2] for angiographic imaging of the renal vasculature in humans [3] and for transstenotic pressure gradients in a swine model [4]. Validation of velocity measurements in vivo with non-MRI methods is desirable, but not possible. The purpose of this study was to assess the repeatability of 2D and 4D-PC flow measurements in humans and to assess the internal consistency of arterial in-flow and out-flow measurements in the renal vasculature.

## Methods

**Figure 1:**
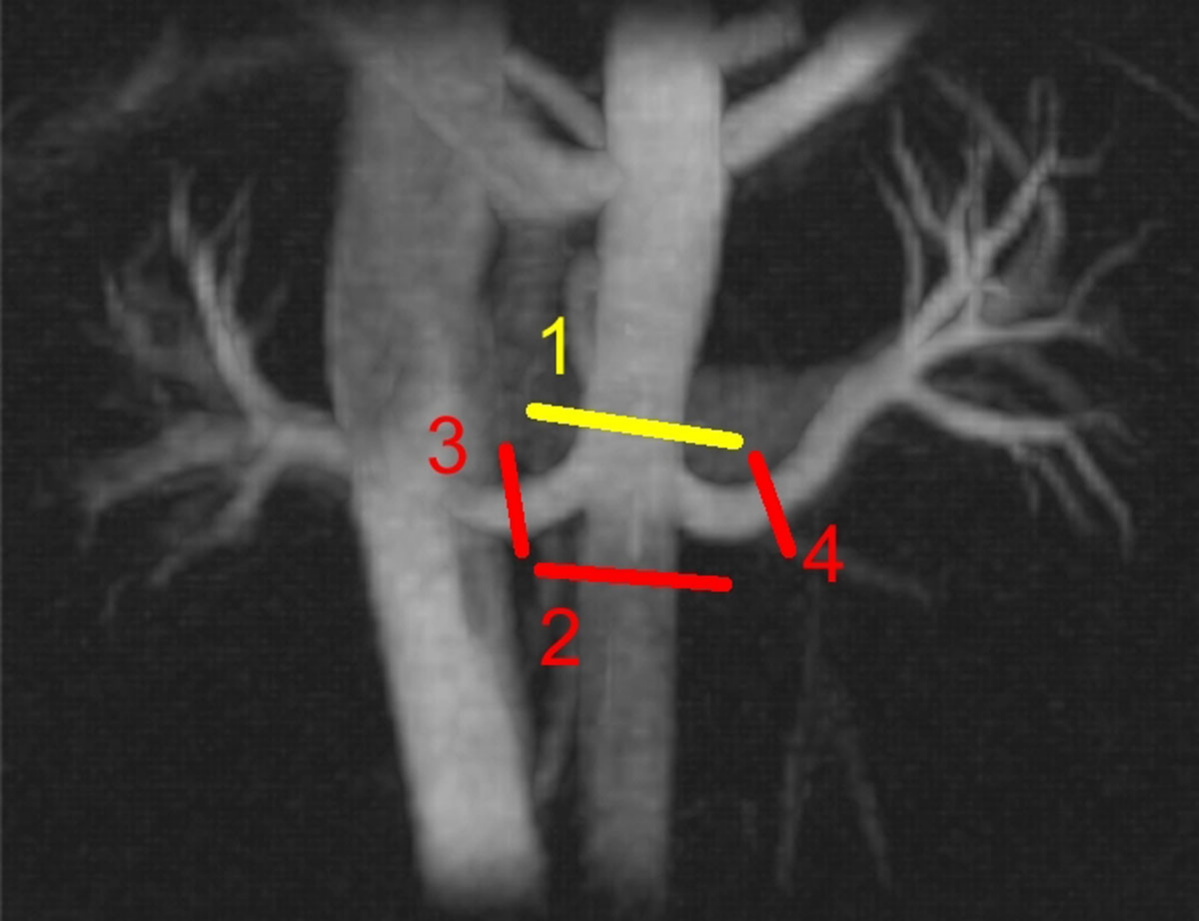
MIP from the complex difference data of a PC-VIPR data set. For the 2D scans, slices were acquired in the suprarenal aorta (1), infrarenal aorta (2), and right (3) and left (4) renal arteries. Flow measurements from the PC VIPR data were taken at the same locations as the 2D acquisitions. Arterial in-flow (Q_in_) is shown in yellow; arterial out-flow (Q_out_) is shown in red.

Eight healthy volunteers (mean age=26.9±2.5years) were scanned on a 3TMR scanner with a 32-channel phased-array torso coil. Subjects refrained from eating for a minimum of four hours prior to the MR examination. 2DPC scan planes were prescribed in the supra- and infrarenal aorta and in each renal artery (Fig.1) with a double-oblique orientation. A radially undersampled 4D-PC technique (PC-VIPR) with large volume coverage over the abdomen and 3-directional velocity encoding was also acquired. Each subject then got off of the scanner bed for five minutes and subsequently returned to repeat the MR protocol.

Total flow over the cardiac cycle was measured with in-house-developed software. To test for internal consistency, differences were computed between the suprarenal aortic flow (Q_in_) and the sum of flow measurements in the renal arteries and infrarenal aorta (Q_out_); differences were normalized by the average of Q_in_ and Q_out_. 2D and 4D percent differences were compared with a Student's t-test. The repeatability of flow measurements was assessed with Pearson correlation and Bland-Altman analysis.

## Results

**Figure 2:**
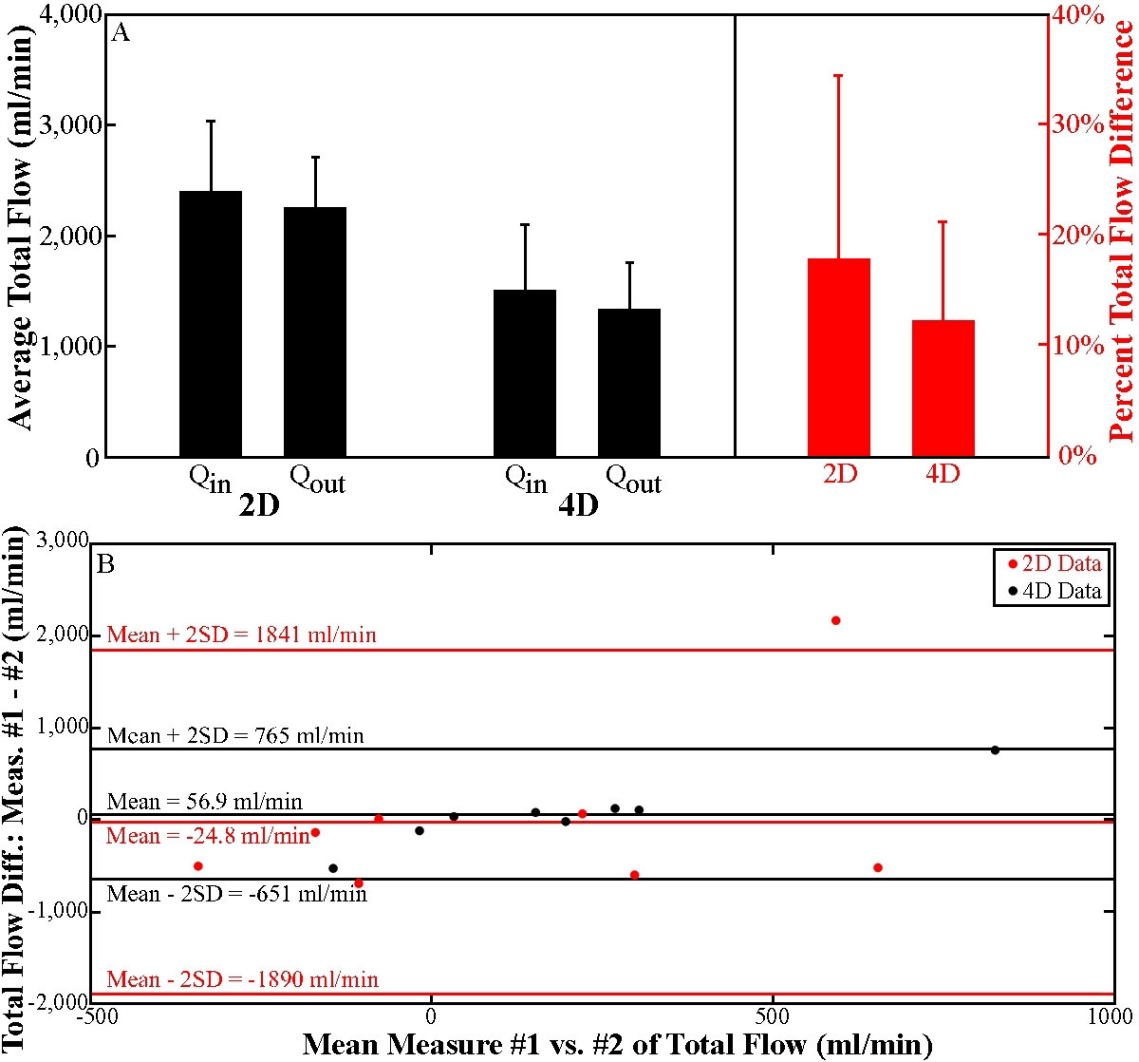
**A.** Average absolute and percent total flow differences for 2D and 4D PC flow measurements. Flow differences were computed as the difference between suprarenal aortic flow (Q_in_) and the sum of the flow in the infrarenal aorta and two renal arteries (Q_out_). Measurements were normalized by the average of these two values. Bars are standard deviation. **B.** Bland-Altman plots for the repeatability of 2D and 4D flow difference measurements.

For all 16 sets of 2D measurements, average Q_in_ (2404.8±632.5ml/min) was similar to average Q_out_ (2259.1±449.4ml/min). Similarly, for all 16 sets of 4D measurements, average Q_in_ (1517.5±585.2ml/min) was similar to average Q_out_ (1343.9±410.8ml/min). 4D flow measurements tended to be lower in magnitude than 2D flow measurements. Normalized, the total flow difference for all 16 2D sets of measurements (18.0±16.5%) was greater than the mean percent difference of all 16 4D measurements (12.4±8.8%;p=0.053;Fig.2A). The repeatability of both 2D (r=0.91,p<1x10^-12^) and 4D (r=0.86,p<1x10^-9^) flow measurements was strongly correlative. Bland-Altman analysis demonstrated small overall flow differences for both the 2D (mean±2SD=-24.8±1865ml/min) and 4D (mean±2SD=56.9±708ml/min) techniques (Fig.2B).

## Conclusions

Flow measurements in healthy volunteers revealed that 4D measurements tended to be more internally consistent than 2D measurements, with average differences slightly greater than 10%. The repeatability of the 2D and 4D data were similar. These results are favorable compared to a previous report [5] comparing 2D and 4D PC flow measurements, despite the compounding error of multiple flow measurements for this check on internal consistency.
